# Predictive value of the systemic immune-inflammation index on one-year mortality in geriatric hip fractures

**DOI:** 10.1186/s12877-024-04916-3

**Published:** 2024-04-15

**Authors:** Zekeriya Ersin Çelen

**Affiliations:** Department of Orthopaedics and Traumatology, Ankara Bilkent City Hospital, Ankara, Turkey

**Keywords:** Systemic immune-inflammation index, Mortality, Survival, Hip fracture, Elderly, Risk factor

## Abstract

**Background:**

Geriatric hip fractures are associated with a high incidence of mortality. This study examines the predictive value of the systemic immune-inflammation index (SII) on one-year mortality in elderly hip fracture patients.

**Methods:**

A single-center retrospective study was conducted between February 2017 and October 2020. Three hundred and eleven surgically treated consecutive hip fracture patients were included in the study. Admission, postoperative first day, and postoperative fifth-day SII values were calculated. The receiver operating characteristic (ROC) curve was used to calculate the cut-off values, and patients were divided into high and low groups according to these cut-off values. After univariate Cox regression analysis, significant factors were included in the multivariate Cox proportional hazards model to adjust the effect of covariates and explore independent predictive factors associated with mortality. Further subgroup analysis was performed to evaluate the accuracy of the results for different clinical and biological characteristics.

**Results:**

The mean age was 80.7 ± 8.0 years, and women made up the majority (67.8%) of the patients. The one-year mortality rate was 28.0%. After univariate and multivariate analyses, high postoperative fifth-day SII remained an independent predictor of one-year mortality (adjusted HR 2.16, 95% CI 1.38–3.38, *p* = 0.001). Older age, male gender, Charlson comorbidity index (CCI) ≥ 2, and hypoalbuminemia were found to be other independent predictors. The optimal cut-off value of the postoperative fifth-day SII was calculated at 1751.9 units (*p* < 0.001).

**Conclusion:**

The postoperative fifth-day SII is a simple and useful inflammatory biomarker for predicting one-year mortality in patients with hip fracture.

## Introduction

Since the human life span has increased in recent years, there has been an increase in the elderly population, and geriatric hip fractures have become a global public health problem. Despite the fact that one-year mortality rates for hip fractures have been reduced in the last six decades [1], the expected global number of hip fractures is expected to reach 4.5 million by the year 2050 [2]. Therefore, it is important to reveal the predictive factors for mortality.

Several prediction models have been developed to predict mortality after hip fractures, such as the Orthopaedic Physiological and Operative Severity Score (O-POSSUM), the Estimation of Physiologic Ability and Surgical Stress (E-PASS), and the Nottingham Hip Fracture Score (NFMS) [3, 4]. Nevertheless, none of the existing models provided excellent discrimination [5, 6].

Several recent studies have focused on inflammatory biomarkers that may predict mortality in geriatric hip fractures. In rat models, systemic inflammation has been shown to be aggravated by hip fracture in aged animals [7]. Consistently, clinical studies have also demonstrated that inflammatory biomarkers such as TNF-α, IL-6, IL-10, and CRP may play an important role in postoperative mortality in elderly hip fracture patients [8, 9].

Systemic immune-inflammation index (SII) is a simply available inflammatory biomarker that can be easily calculated using the formula plateletxneutrophil/lymphocyte counts. This marker has been found to be associated with the prognosis of many malignancies, cardiac diseases, and cerebrovascular events [10–12]. Furthermore, another study reported an increased osteoporotic fracture risk in postmenopausal osteoporosis patients with high SII [13]. Additionally, a recent study found a relationship between high admission SII and low survival rates in hip fracture patients [14]. However, in that study, the effect of SII on mortality was evaluated only preoperatively. The current study aims to examine the prognostic value of the SII not only immediately after hospital admission but also in the postoperative first and fifth days to evaluate the effects of persisting inflammation.

## Materials and methods

**Fig. 1:**
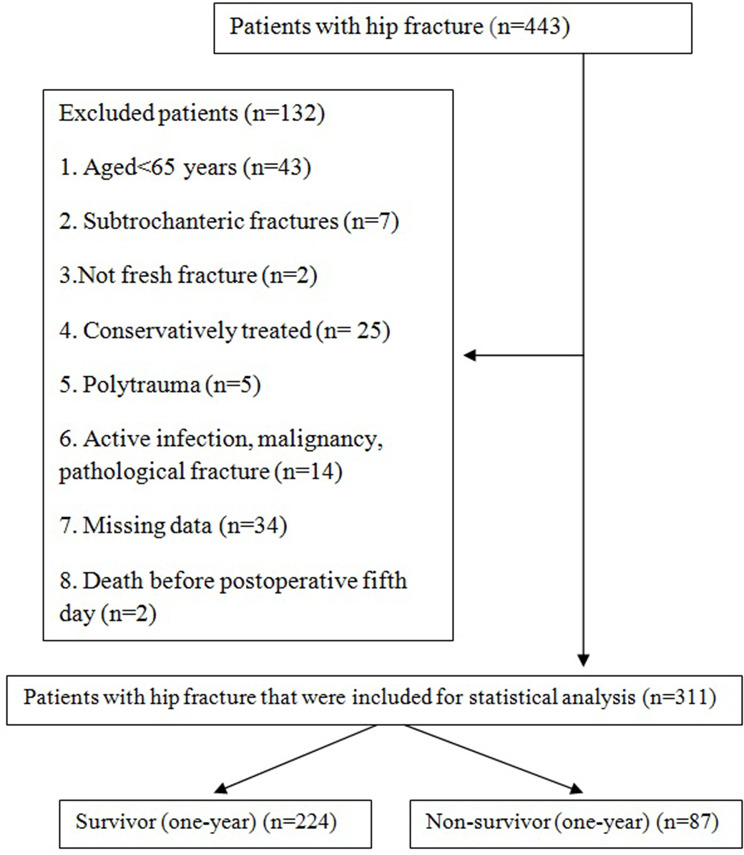
Patient flow chart diagram

A total of 443 hip fracture patients who applied to a state hospital between February 2017 and October 2020 were evaluated retrospectively. The inclusion criteria were being over 65 years of age and having been operated on for a hip fracture. Because we investigated an inflammatory biomarker, we excluded conditions that could disrupt the inflammatory status of the patient such as active infection or malignancy. Additionally, as this study focuses on geriatric hip fractures, we excluded some fracture types and several factors to decrease heterogeneity. Therefore, patients with subtrochanteric fracture, not fresh fracture (≥ 3 weeks), polytrauma, active infection, malignancy, pathological fracture, missing data, and conservatively treated patients were excluded. In addition, two patients who died before the fifth day were excluded from this study. A chart of inclusions is shown in Fig. 1.

The study was approved by the Institutional Ethics Committee (IRB number: 2021/136), and written informed consent was obtained from patients or their immediate family members.

### Data collection

Patient characteristics were collected from the hospital database, including age, gender, admission date, operation date, American Society of Anaesthesiologists (ASA) score, comorbidities, fracture type (femur neck or intertrochanteric fracture), treatment details (arthroplasty or internal fixation), blood transfusion, anesthesia type (general or spinal), duration of stay in the intensive care unit (ICU), albumin value, hematological markers from the complete blood count, and differential counts of leukocytes. Blood samples were analyzed using an automated blood cell counter (Mindray BC-6800, Shenzhen, China). The survival status and death dates of the patients were obtained from the national death notification system. Causes of death were not available.

Admission (SII0), postoperative first day (SII1), and postoperative fifth day SII (SII5) values were calculated using the formula neutrophilxplatelet/lymphocyte counts as defined previously and expressed as x 10^9^/L [15]. Anemia was defined as < 13.0 g/dL in men and < 12.0 g/dL in women, according to World Health Organization (WHO) criteria. The reference range of albumin was between 3.5 and 5.5 g/dL. Albumin levels were classified as < 3.5 g/dL (hypoalbuminemia) and ≥ 3.5 g/dL (normal albumin). No patient exceeded the upper albumin limit (> 5.5). The comorbidities of the patients were assessed using the CCI (Charlson Comorbidity Index), which includes 17 comorbid conditions and assigns 1 to 6 points for each comorbidity according to its impact on mortality [16]. Based on previous studies, CCI was categorized as none (CCI = 0), low (CCI = 1), and high (CCI ≥ 2) [17].

### Perioperative care

A perioperative dose of cefazolin was administered to the patients 30–60 min before surgery and 2 g throughout the 48 h postoperatively. Subcutaneous low-molecular-weight heparin (4000 IU, 1 × 1) was given 12 h before surgery and throughout the postoperative 4 weeks. Patients were mobilized on the first postoperative day with the aid of a walker under the guidance of a physiotherapist.

### Statistical analysis

The data were included in a database created by the Excel 2007 program by Microsoft (Microsoft Corporation, Redmond, Washington, USA). Statistical analysis was performed using PASW statistics for Windows (version 18, USA). The Shapiro-Wilk test was used to evaluate normal distribution. Continuous variables were expressed as means ± standard deviation (SD) or median and interquartile range (IQR) according to the distribution. Differences between the groups were evaluated using the Student’s t test for normally distributed variables and the Mann-Whitney U test for the variables not distributed normally. Categorical variables were described as frequencies and compared using the chi-square test. A receiver operating characteristic (ROC) curve was generated to determine the cut-off values for the SII0, SII1, and SII5; then patients were divided into high and low groups for each variable. The Kaplan-Meier method was used to estimate the survival rates, and the log rank test was used to evaluate the differences between groups. After adjusting for significant variables identified by the univariate Cox regression analysis, a further multivariate Cox proportional hazards model was generated to identify the independent risk factors for mortality. Because of the collinearity with the variable CCI, the ASA (American Society of Anesthesiologists) score was not introduced in the Cox regression model. Subgroup analyses were also performed for each stratified group to further investigate whether the predictive value of SII was consistent among different populations. A p value of < 0.05 was considered statistically significant.

## Results

Of the 443 patients treated for hip fractures, 311 were evaluated in the study according to the inclusion and exclusion criteria. The patient characteristics are summarized in Table 1. The mean age was 80.7 ± 8.0 years, and women constituted the majority (67.8%) of the patients. The method of anesthesia was spinal anesthesia for 94.2% of patients. The proportions of none (CCI = 0), low (CCI = 1), and high (CCI ≥ 2) comorbidity were 14.8%, 26.4%, and 58.8%, respectively. The majority (76.2%) of the patients were treated with arthroplasty. Laboratory data showed that the median values of SII0, SII1, and SII5 were 1557.7, 1767.1, and 1558.1 units, respectively. The mean hospitalization duration was 11.3 ± 8.1 days. After one year of follow-up, 87 (28.0%) of the patients died.

**Table 1:** Baseline characteristics and outcomes of the study patients

Characteristics	Total (*N* = 311)
Age, years (mean, SD)	80.7 ± 8.0
Female gender, n(%)	211 (67.8%)
CCI, n (%)	
CCI = 0	46 (14.8%)
CCI = 1	82 (26.4%)
CCI ≥ 2	183 (58.8%)
Anesthesia type (spinal/genel)	293 (94.2%) /18 (5.8%)
ASA score (mean, SD)	2.9 ± 0.5
ICU stay duration, days (mean, SD)	1.6 ± 4.8
Fracture type, n (%)	
Neck	107 (34.4%)
Intertrochanteric	204 (65.6%)
Surgery type, n(%)	
Arthroplasty	237 (76.2%)
Internal fixation	74 (23.8%)
Delay to surgery, days (mean, SD)	3.4 ± 2.3
Albumin (median, IQR)	3.6 (3.2–3.9)
Hemoglobin (median, IQR)	11.5 (10.3–12.6)
SII0, units (median, IQR)	1557.7 (807.8-2720.9)
SII1, units (median, IQR)	1767.1 (1077.3-2702.1)
SII5, units (median, IQR)	1558.1 (984.7-2390.5)

CCI: Charlson comorbidity index, ASA: American society of anaesthesiologists, ICU: Intensive care unit, SII: Systemic immune-inflammatory index

**Fig. 2:**
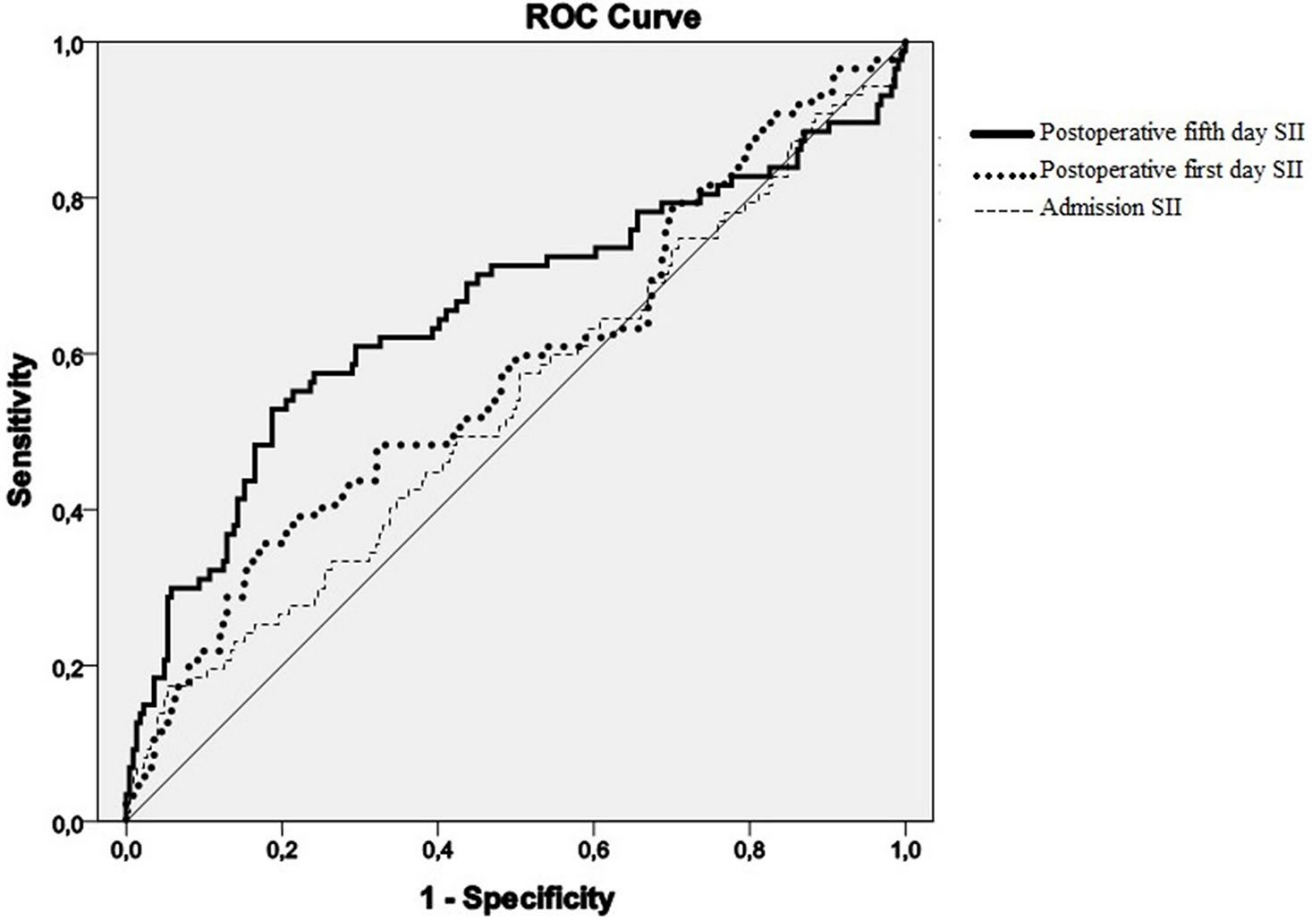
Receiver operating characteristics (ROC) curves of systemic immune-inflammation index (SII) for predicting one-year survival. Area under curve (AUC) of postoperative fifth day SII curve (AUC 0.66, 95% CI 0.58–0.73) was higher than that of admission (AUC 0.54, 95%CI 0.46–0.61) and postoperative first day (AUC 0.58, 95% CI 0.50–0.65) values

According to the ROC analysis, the optimal cut-off value for predicting one-year mortality was calculated as 1572.7 units for SII0, 1794.1 units for SII1, and 1751.9 units for SII5. The area under the curve (AUC) of SII5 (AUC 0.66, 95% CI 0.58–0.73, sensitivity 62.1, specificity 67.4, *p* < 0.001) was larger than SII0 (AUC 0.54, 95% CI 0.46–0.61) and SII1 (AUC 0.58, 95% CI 0.50–0.65) (Fig. 2).

**Fig. 3:**
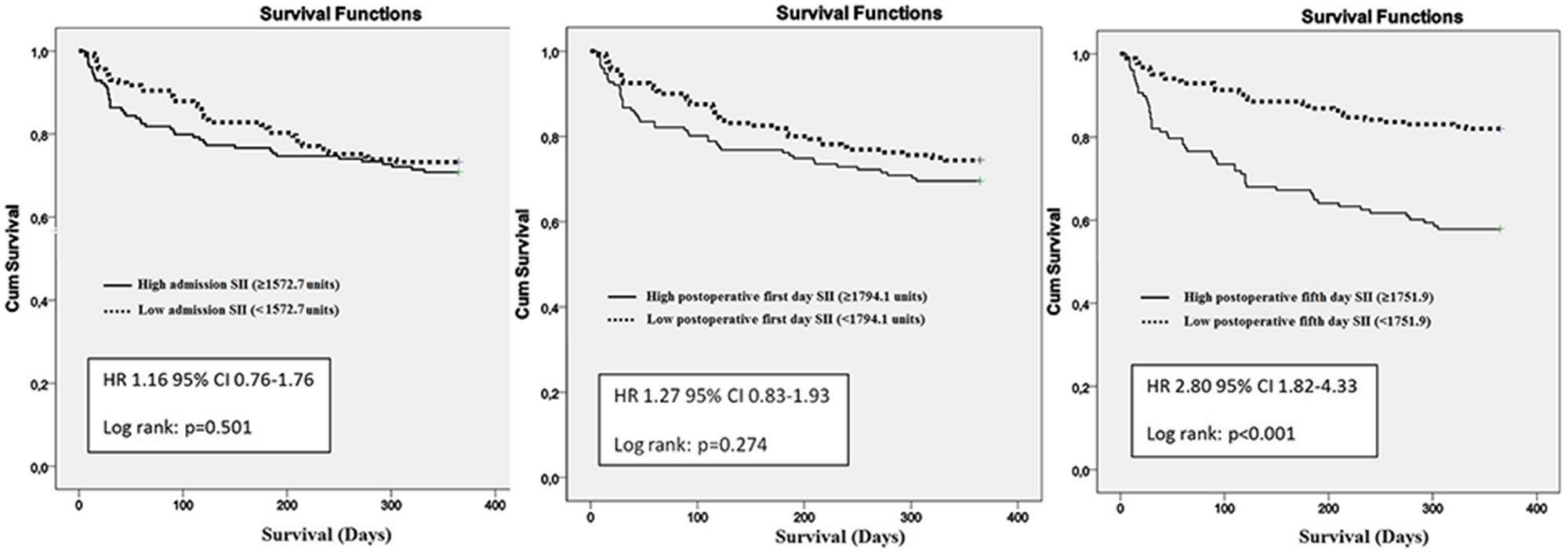
Kaplan Meier survival analyses of admission, postoperative first day and postoperative fifth day systemic immune-inflammation index (SII). Hazard ratio (HR) was estimated using Cox regression analysis. P value was calculated using log-rank test

In univariate analysis, older age, male gender, CCI ≥ 2, delay to surgery, hypoalbuminemia, and high (≥ 1751.9) SII5 levels were significantly associated with one-year mortality (Table 2). The log-rank test of the Kaplan-Meier curves indicated that the patients with high SII5 levels had worse one-year survival (*p* < 0.001) (Fig. 3).

**Table 2:** Univariate and multivariate Cox regression analysis of risk factors associated with one-year mortality

Variables	Univariate		Multivariate	
	HR (95% CI)	*P* value	HR (95% CI)	*P* value
Age (per 1 year increase)	1.09 (1.05–1.12)	**< 0.001**	1.07 (1.04–1.11)	**< 0.001**
Gender (male vs. female)	1.75 (1.15–2.68)	**0.009**	1.68 (1.08–2.61)	**0.020**
CCI = 0	1.0 (Reference)	-	1.0 (Reference)	-
CCI = 1	1.87 (0.68–5.09)	0.223	1.89 (0.68–5.23)	0.222
CCI = 2	3.82 (1.54–9.49)	**0.004**	3.15 (1.25–7.91)	**0.015**
Fracture type (ITF vs. neck)	1.05 (0.68–1.63)	0.821	-	-
Surgery type (fixation vs. arthroplasty)	0.96 (0.58–1.58)	0.875	-	-
**Delay to surgery (> 48 h vs. ≤ 48 h)**	1.76 (1.10–2.82)	**0.018**	1.52 (0.94–2.44)	0.087
Blood transfusion (yes vs. no)	1.31 (0.82–2.09)	0.264	-	-
Anemia (yes vs. no)	1.58 (0.97–2.59)	0.067	-	-
**Albumin (< 3.5 vs. ≥ 3.5)**	2.19 (1.43–3.35)	**< 0.001**	1.71 (1.11–2.64)	**0.016**
SII0 (high group vs. low group), 10^9^/L	1.16 (0.76–1.76)	0.501	-	-
SII1 (high group vs. low group), 10^9^/L	1.27 (0.83–1.93)	0.274	-	-
SII5 (high group vs. low group), 10^9^/L	2.80 (1.82–4.33)	**< 0.001**	2.16 (1.38–3.38)	**0.001**

HR: Hazard ratio, CCI: Charlson comorbidity index, ITF: Intertrochanteric fracture, SII: Systemic immune-inflammation index

After inclusion of significant factors in the multivariate analysis to adjust the effects of covariates, high SII5 (adjusted HR 2.16, 95% CI 1.38–3.38) remained an independent prognostic factor for predicting mortality (*p* = 0.001). In addition, older age (HR 1.07, 95% CI 1.04–1.11, *p* < 0.001), male gender (HR 1.68, 95% CI 1.08–2.61, *p* = 0.020), CCI ≥ 2 (HR 3.15, 95% CI 1.25–7.91, *p* = 0.015), and hypoalbuminemia (HR 1.71, 95% CI 1.11–2.64, *p* = 0.016) were also independent risk factors for one year mortality (Table 2).

Further subgroup analysis was performed based on different patient characteristics. In most stratified groups, consistent with the main analysis, SII5 was significantly associated with one-year mortality except for only one subgroup, including patients with CCI = 0 (*p* = 0.223) (Table 3).

**Table 3:** Subgroup analysis estimating the association between the postoperative fifth day systemic immune-inflammation index and one-year mortality based on different stratified groups

Subgroups	HR (95% CI)	*P* value
**Age**		
< 80 years	3.73 (1.47–9.47)	**0.006**
≥ 80 years	2.29 (1.40–3.74)	**0.001**
**Gender**		
Female	2.94 (1.65–5.23)	**< 0.001**
Male	2.42 (1.25–4.68)	**0.009**
**CCI**		
CCI = 0	1.07 (1.03–1.12)	0.223
CCI = 1	3.37 (1.22–9.29)	**0.019**
CCI ≥ 2	2.36 (1.44–3.85)	**0.001**
**Albumin**		
Hypoalbuminemia	3.26 (1.82–5.85)	**< 0.001**
Normal albumin	2.22 (1.15–4.29)	**0.018**
**Fracture type**		
Intertrochanteric	3.07 (1.78–5.31)	**< 0.001**
Neck	2.35 (1.15–4.79)	**0.019**
**Surgery type**		
Arthroplasty	2.71 (1.68–4.38)	**< 0.001**
Internal fixation	3.01 (1.07–8.47)	**0.034**
**Delay to surgery**		
> 48 h	2.22 (1.35–3.66)	**0.002**
≤ 48 h	5.37 (2.13–13.54)	**< 0.001**
**Hemoglobin**		
Anemia	2.78 (1.17–6.66)	**0.021**
Normal hemoglobin	2.72 (1.64–4.49)	**< 0.001**

HR: Hazard ratio, CCI: Charlson comorbidity index

## Discussion

The most important finding of the current study was that postoperative fifth-day SII was an independent predictive factor for mortality in geriatric hip fracture patients (*p* = 0.001). A cut-off value of ≥ 1751.9 SII5 was calculated with a 2.16-fold increased risk in the multivariate Cox proportional hazards model (*p* = 0.001). Recently, a study reported that admission SII may be a good predictive factor for elderly hip fractures [14]. However, the limitation of that study was the absence of postoperative SII evaluations. Moreover, the study reported a 9% one-year mortality rate, which is extremely low compared to the contemporary literature. In the current study cohort, ROC analyses were performed for SII0, SII1, and SII5. It was observed that the prognostic value of the SII was clearly more prominent days after surgery, and the highest area under curve was observed in the SII5 curve. In addition, in univariate and multivariate analyses, SII5 was found to be an independent risk factor rather than SII0 and SII1.

A more recent study evaluated the predictive value of preoperative and postoperative SII on mortality in hip hemiarthroplasty patients [18]. The multivariate analysis of the study showed borderline significance for SII5 (*p* = 0.055). Although the study couldn’t find a discriminatory ability for the laboratory parameters investigated, it was concluded that these parameters could help develop early therapeutic interventions to improve patient outcomes. The reason why the study failed to find a significant predictive effect for SII5 may be because of the small sample size. In addition, consistent with our study, the study found no significant predictive value of admission SII on one-year mortality rates [18].

In a previous registry study, a one-year mortality rate of 30.7% was reported in patients who underwent surgery following a fracture of the hip [19]. The one-year mortality rate of the current study was 28.0%, which is consistent with the previous literature. High CCI has previously been reported as a risk factor for poor survival in elderly hip fractures [14, 20]. In the current study, high CCI (≥ 2) was found to be associated with a 3.15-fold higher one-year mortality rate in the multivariate analysis (*p* = 0.015). Older age and male gender have also been reported as associated with increased mortality in patients with hip fractures [20]. In the current patient cohort, each 1-year increase in age increased the risk of one-year mortality by 7% (*p* < 0.001). In addition, male gender was found to be associated with a 1.68-fold higher one-year mortality rate (*p* = 0.020).

Hypoalbuminemia has also been shown to be a risk factor for poor survival in geriatric hip fractures [21]. In the current study, hypoalbuminemia was observed to be associated with a 1.71-fold increase in one-year mortality (*p* = 0.016). Early surgery within the first 48 h has been shown to be associated with lower mortality rates in patients with hip fractures [22]. In the current study, consistent with the literature, a > 48-hour delay to surgery was associated with a higher mortality risk in the univariate analysis (HR: 1.76, *p* = 0.018). However, delay to surgery lost statistical significance in the multivariate analysis, but there was a trend toward significance (*p* = 0.087).

Early identification of risk factors and implementation of preventive measures may be beneficial in reducing postoperative mortality rates after hip fracture surgery. According to our results, older age, male gender, high CCI, hypoalbuminemia, and high postoperative fifth-day SII seem to be reliable predictors of postoperative mortality in these patients. These parameters can be used for individualized perioperative management and to stratify the risk of postoperative mortality. Monitoring postoperative fifth-day SII, proper management of comorbidities perioperatively, and prevention of hypoalbuminemia with appropriate nutritional support are strategies that might be applied in order to optimize their outcomes.

Various predictive models have previously been developed that can predict mortality after hip fractures. However, a recent meta-analysis reported strong evidence that O-POSSUM and P-POSSUM cannot accurately predict postoperative mortality among such patients [4]. Another study from Sweden reported that the POSSUM score and NHFS showed poor discrimination in hip fracture patients, and mortality was largely dependent on parameters not included in these scores [6]. Furthermore, one study evaluated six different prediction models, including CCI, O-POSSUM, E-PASS, and NHFS. Despite these methods combined a variety of parameters ranging from six to nineteen parameters, none of them contained SII, and none of them provided excellent discrimination [5]. In our study, postoperative fifth-day SII alone provided moderate discrimination without being combined with any other parameters. Since SII5 is a biomarker that can be calculated relatively easily, it can be performed quickly in clinical practice. In addition, this biomarker can be combined with other predictive models to increase its discriminative ability in clinical practice. Future predictive models might combine SII5 with different models or patient characteristics.

This study has several limitations. First, the current study used data obtained from retrospective screening, and some variables were not obtained that could affect survival and inflammatory markers, such as body mass index, risk of fall assessment, injury hospitalization interval, and smoking. Second, due to the fact that many surgeons performed the surgeries, potentially there may be some performance bias in the study. Third, some of the subgroups had relatively small patient numbers, which limited statistical analysis in these subgroups. Fourth, the study was based on a single-center retrospective design, which might limit the generalizability of the findings. Lastly, although a higher AUC was observed in SII5 than in SII0 and SII1, the AUC value was lower than the threshold value of 0.70, indicating moderate discriminatory ability. However, Kaplan-Meier survival curves, log rank tests, and the multivariate models demonstrated the ability of SII5 to predict one-year mortality. Considering the retrospective nature of the analysis, the results need to be validated by prospective cohort studies.

In conclusion, elevated (≥ 1751.9 units) postoperative fifth-day SII is associated with increased one-year mortality in patients with hip fractures. As SII is a simple and economical marker that can be calculated from a routine blood test, it can be easily performed in usual clinical practice for risk stratification of mortality in this patient population.

## Data Availability

The datasets used and/or analysed during the current study available from the corresponding author on reasonable request.
